# Supplementary material for: Dynamic de novo heterochromatin assembly and disassembly at replication forks ensures fork stability
Source: Nat Cell Biol. 2023 Jul 6;25(7):1017–32. doi: 10.1038/s41556-023-01167-z (PMC10344782; doi:10.1038/s41556-023-01167-z)
Supplement: Supplementary file 1 — Reporting Summary [file 41556_2023_1167_MOESM1_ESM.pdf]

nature portfolio

Corresponding author(s): Nitika Taneja

Last updated by author(s): May 10, 2023

Reporting Summary

Nature Portfolio wishes to improve the reproducibility of the work that we publish. This form provides structure for consistency and transparency in reporting. For further information on Nature Portfolio policies, see our Editorial Policies and the Editorial Policy Checklist.

Statistics

For all statistical analyses, confirm that the following items are present in the figure legend, table legend, main text, or Methods section.

n/a	Confirmed	
☐	☒	The exact sample size (*n*) for each experimental group/condition, given as a discrete number and unit of measurement
☒	☐	A statement on whether measurements were taken from distinct samples or whether the same sample was measured repeatedly
☐	☒	The statistical test(s) used AND whether they are one- or two-sided<br>*Only common tests should be described solely by name; describe more complex techniques in the Methods section.*
☐	☒	A description of all covariates tested
☒	☐	A description of any assumptions or corrections, such as tests of normality and adjustment for multiple comparisons
☐	☒	A full description of the statistical parameters including central tendency (e.g. means) or other basic estimates (e.g. regression coefficient) AND variation (e.g. standard deviation) or associated estimates of uncertainty (e.g. confidence intervals)
☐	☒	For null hypothesis testing, the test statistic (e.g. *F*, *t*, *r*) with confidence intervals, effect sizes, degrees of freedom and *P* value noted<br>*Give P values as exact values whenever suitable.*
☒	☐	For Bayesian analysis, information on the choice of priors and Markov chain Monte Carlo settings
☒	☐	For hierarchical and complex designs, identification of the appropriate level for tests and full reporting of outcomes
☐	☒	Estimates of effect sizes (e.g. Cohen's *d*, Pearson's *r*), indicating how they were calculated

Our web collection on statistics for biologists contains articles on many of the points above.

Software and code

Policy information about availability of computer code

Data collection	Illumina HiSeq2000, Metafer 5 , Leica ST5 confocal microscope
Data analysis	Graphpad Prism 9.4.1, imageJ 1.53t, Metasystem, R studio 1.2.5019, Flowjo v10.8.1, Spotfire Analyst 12.0, FastQC v0.11.7, Proteome Discoverer (version 2.5.0.400)

For manuscripts utilizing custom algorithms or software that are central to the research but not yet described in published literature, software must be made available to editors and reviewers. We strongly encourage code deposition in a community repository (e.g. GitHub). See the Nature Portfolio guidelines for submitting code & software for further information.

Data

Policy information about availability of data

All manuscripts must include a data availability statement. This statement should provide the following information, where applicable:

- Accession codes, unique identifiers, or web links for publicly available datasets
- A description of any restrictions on data availability
- For clinical datasets or third party data, please ensure that the statement adheres to our policy

Deep-sequencing (ChIP-seq, and RNA–seq) data that support the findings of this study have been deposited as a Bioproject under accession code PRJNA845122, for the RNA-sequencing data, and PRJNA897702, for the ChIP-sequencing data.
Mass spectrometry data have been deposited in ProteomeXchange with the primary accession codes PXD041742, for silac data and PXD041914 for the proteomics

analysis of histone PTM levels
The human ovarian cancer data analyzed in this study were from the TCGA datasets102,103 (https://link.springer.com/article/10.1007/s11357-023-00742-4/tables/1).
Source data have been provided in Source Data. All other data supporting the findings of this study are available from the corresponding author on reasonable request.

Human research participants

Policy information about studies involving human research participants and Sex and Gender in Research.

Reporting on sex and gender	N/A
Population characteristics	N/A
Recruitment	N/A
Ethics oversight	N/A

Note that full information on the approval of the study protocol must also be provided in the manuscript.

Field-specific reporting

Please select the one below that is the best fit for your research. If you are not sure, read the appropriate sections before making your selection.

☒ Life sciences ☐ Behavioural & social sciences ☐ Ecological, evolutionary & environmental sciences

For a reference copy of the document with all sections, see nature.com/documents/nr-reporting-summary-flat.pdf

Life sciences study design

All studies must disclose on these points even when the disclosure is negative.

Sample size	N/A. Sample size for samples was chosen according to or exceeding standards in the field
Data exclusions	N/A, no data exclusion
Replication	Experimental assays were performed two - three independent replicates, with similar results.
Randomization	This is not applicable, as our sample groups were grown under the same condition and collect randomly when given treatment without any bias.
Blinding	Blinding was not needed as data is collected by imaging software which yield unbiased, objective measurments

Reporting for specific materials, systems and methods

We require information from authors about some types of materials, experimental systems and methods used in many studies. Here, indicate whether each material, system or method listed is relevant to your study. If you are not sure if a list item applies to your research, read the appropriate section before selecting a response.

Materials & experimental systems

n/a	Involved in the study	
☐	☒	Antibodies
☐	☒	Eukaryotic cell lines
☒	☐	Palaeontology and archaeology
☒	☐	Animals and other organisms
☒	☐	Clinical data
☒	☐	Dual use research of concern

Methods

n/a	Involved in the study	
☐	☒	ChIP-seq
☐	☒	Flow cytometry
☒	☐	MRI-based neuroimaging

Antibodies

Antibodies used	Anti-BrdU (Clone B44)(347580, BD Bioscience),<br>Anti-Chk1, (G4) (sc8408, Santa Cruz Biotechnology),

	Anti-Chk1 (DCS-310,(Sørensen et al. 2003))<br>Anti-BARD1(A300-263A, Bethyl),<br>BRCA1 (D-9)(SC6954, Santa Cruz Biotechnology),<br>Anti-RPA32/RAP2 [9H8] (Ab2175, Abcam),<br>Anti-BrdU [BU1/75 (ICR1)] (ab6326, Abcam),<br>Anti-PCNA [PC10] (ab29, Abcam),<br>Anti-H3K9me1 [EPR16989] (Ab176880, Abcam),<br>Anti-H3K9me2 (Ab1220, Abcam),<br>Anti-H3K9me3 [EPR16601](Ab176916, Abcam),<br>Anti-G9a [EPR18894](Ab 185050, Abcam),),<br>Anti-H4K20me0 [EPR22116] (Ab227804, Abcam),<br>Anti-H4K16ac [EPR1004] (Ab109463, Abcam),<br>Anti-Phospho-Chk1 (Ser345) (133D3)(#2348, Cell Signaling),<br>Anti-H3 (Ab1791, Abcam),<br>Anti-H3 [1B1B2] (Ab195277, Abcam),<br>Anti-HDAC1 (Ab19845, Abcam),<br>Anti-RAD51 (70-002, Bio Academia),<br>Anti-H2AK15ub (EDL H2AK15-4) (MABE1119, Millipore),<br>Anti-γ H2AX (clone JBW301)(05-636, Millipore),<br>Anti-phospho-H3S10 (06-570, Millipore),<br>Anti-H3K9me3 (Ab8898, Abcam),<br>Anti-H3 (Ab10799, Abcam),<br>Anti-H3K9me3 (07-442, Millipore),<br>Anti-H3K9me1 (Upstate, 07-450),<br>Anti-p53-S15p (D4S1H) (12571, Cell Signaling),<br>Anti-p53 (clone DO-1) (MABE327, Sigma-Aldrich),<br>Anti-b-actin (clone AC-74) (A5316, Sigma-aldrich),<br>Anti-ssDNA antibody (AB_10805144, DSHB)<br>Anti-Biotin antibody (A150-109A, Bethyl Laboratories)<br>Anti-Biotin antibody (AB_2339006, JacksonImmunoResearch)
Validation	All antibodies used are commercially available and have been validated by the manufacturers.

Eukaryotic cell lines

Policy information about cell lines and Sex and Gender in Research

Cell line source(s)	MRC5 sv40 immortalized human fibroblast and mESCs were generated in Nitika Taneja's lab (Lo et al, Science Advances, 2021)<br>Stable TIG-3 human fibroblast was generated in Anja Groth's lab (Alabert et al, Genes Dev, 2015)
Authentication	None of the cell line used were authenticated
Mycoplasma contamination	cell lines are constantly tested for mycoplasma contamination, and were all negative.
Commonly misidentified lines<br>(See ICLAC register)	No commonly misidentified cell lines were used in the study

ChIP-seq

Data deposition

☒ Confirm that both raw and final processed data have been deposited in a public database such as GEO.

☒ Confirm that you have deposited or provided access to graph files (e.g. BED files) for the called peaks.

Data access links<br>*May remain private before publication.*	PRJNA897702
Files in database submission	PRI_D43J_1_0_H3K9_GCCAAT_L006_R1.fastq.gz, PRI_D43J_2_0_H3K9_CTTGTA_L006_R1.fastq.gz,<br>PRI_EETD_1_plus_H3K9_GCCAAT_L008_R1.fastq.gz, PRI_EETD_2_plus_H3K9_CTTGTA_L008_R1.fastq.gz,<br>PRI_QNRF_1_minus_H3K9_GCCAAT_L007_R1.fastq.gz, PRI_QNRF_2_minus_H3K9_CTTGTA_L007_R1.fastq.gz,<br>PRI_CDKD_1_0_H3_GCCAAT_L005_R1.fastq.gz, PRI_CDKD_2_0_H3_CTTGTA_L005_R1.fastq.gz,<br>PRI_FLC4_1_plus_H3_GCCAAT_L007_R1.fastq.gz, PRI_FLC4_2_plus_H3_CTTGTA_L007_R1.fastq.gz,<br>PRI_AXQA_1_minus_H3_GCCAAT_L006_R1.fastq.gz, PRI_AXQA_2_minus_H3_CTTGTA_L006_R1.fastq.gz,
Genome browser session<br>(e.g. UCSC)	https://genome.ucsc.edu/s/nazaret/Gaggioli%20et%20al_2022_initial_submission

Methodology

Replicates	2

Sequencing depth	Provided as a separate file"Gaggioli et al_Sequencing depth information.pdf"
Antibodies	Anti-H3K9me3 (Ab8898, Abcam), Anti-H3 (Ab10799, Abcam)
Peak calling parameters	During analysis of the ChIP seq experiments, peak calling was performed according to the following parameter: Peak detection was performed with MACS2 version 2.0.9 (20111102) using default settings except for parameters '--broad --nomodel --shiftsize=110'. The shift size of 110 bp was calculated as the median over all Phantom Peak.
Data quality	FASTQC, FASTQCScreen
Software	ChIP-seq data are available at the Gene Expression Omnibus (GEO) (PRJNA897702). Raw reads were aligned to the human genome (hg19 assembly excluding non-canonical chromomes i.e. random, unknown and haplotype variant chromosomes) using Bowtie version 0.12.7 with default parameters except '-S -m 1', which excludes reads mapping to multiple chromosomal positions. Bigwig files were generated using the UCSC Kent utilities (Kent et al. 2010).. We allowed only one read per chromosomal position thus eliminating potential spurious spikes, and each remaining read was extended from its 5′-end to a total length of 250 bases, before converting to bedGraph format, scaling to mapped reads per million and final convertion to bigwig format. Individual BigWig files were uploaded to the UCSC browser for visualization(Kent et al. 2002;2010). To generate chromosome-wide landscapes of H3K9me3 and H3 we used the mean as the combining function and a smoothing window of 4 pixels

Flow Cytometry

Plots

Confirm that:

☒ The axis labels state the marker and fluorochrome used (e.g. CD4-FITC).

☒ The axis scales are clearly visible. Include numbers along axes only for bottom left plot of group (a 'group' is an analysis of identical markers).

☒ All plots are contour plots with outliers or pseudocolor plots.

☒ A numerical value for number of cells or percentage (with statistics) is provided.

Methodology

Sample preparation	Cells were grown to 70–80% confluency in a 10cm culturing dish. Cells were labeled with EdU for 30minutes followed by fixation for 10minutes in 4% formaldehyde in PBS at room temperature. Cells were then washed with 1% BSA/PBS and permeabilized in 0.5% saponin buffer in 1% BSA/PBS. Incorporated EdU were labelled with the click-it reaction using Alexa Fluor® 594 azide according to the manufacturer's protocol (Invitrogen). DAPI was used to stain the DNA.
Instrument	BD LSRFortessa Cell Analyzer
Software	Flowjo v10.8.1
Cell population abundance	The single cells population after SSC and FSC gating was around 60%-90% of the total event.
Gating strategy	Gating was done using SSC-A vs FSC-A, followed by FSC-H vs FSC-W and SSC-H vs SSC-W to select single nuclei.

☒ Tick this box to confirm that a figure exemplifying the gating strategy is provided in the Supplementary Information.
