# Supplementary figures and images for: Dynamic de novo heterochromatin assembly and disassembly at replication forks ensures fork stability
Source: Nat Cell Biol. 2023 Jul 6;25(7):1017–32. doi: 10.1038/s41556-023-01167-z (PMC10344782; doi:10.1038/s41556-023-01167-z)

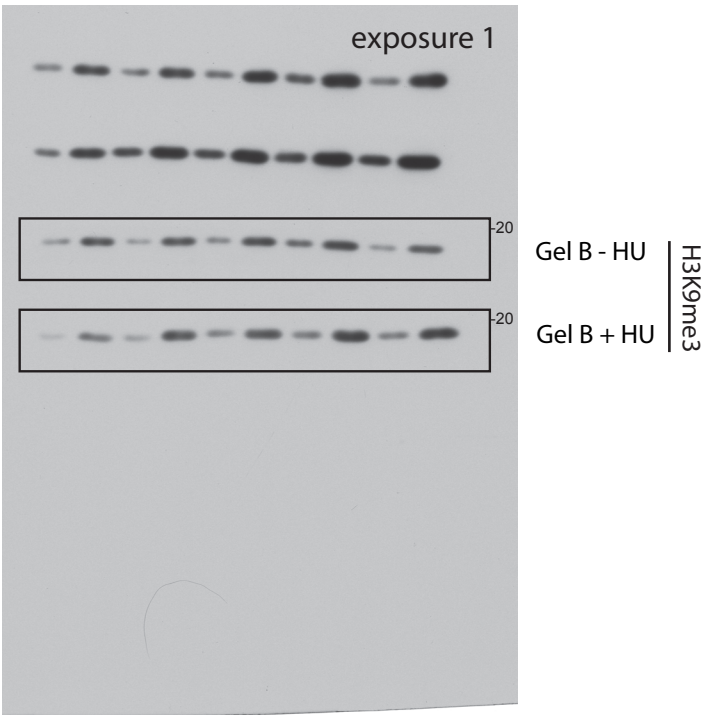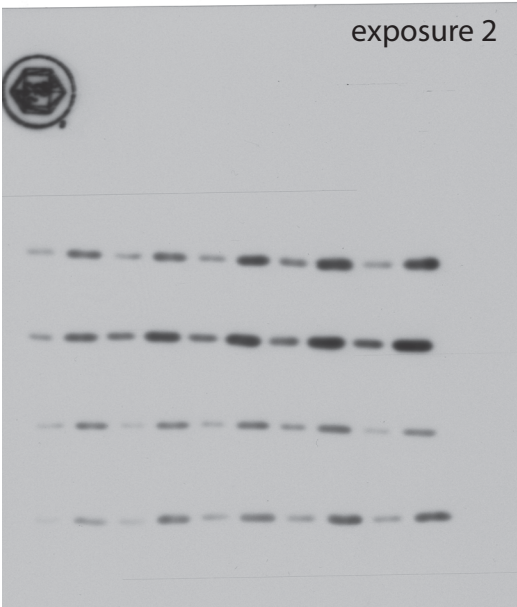

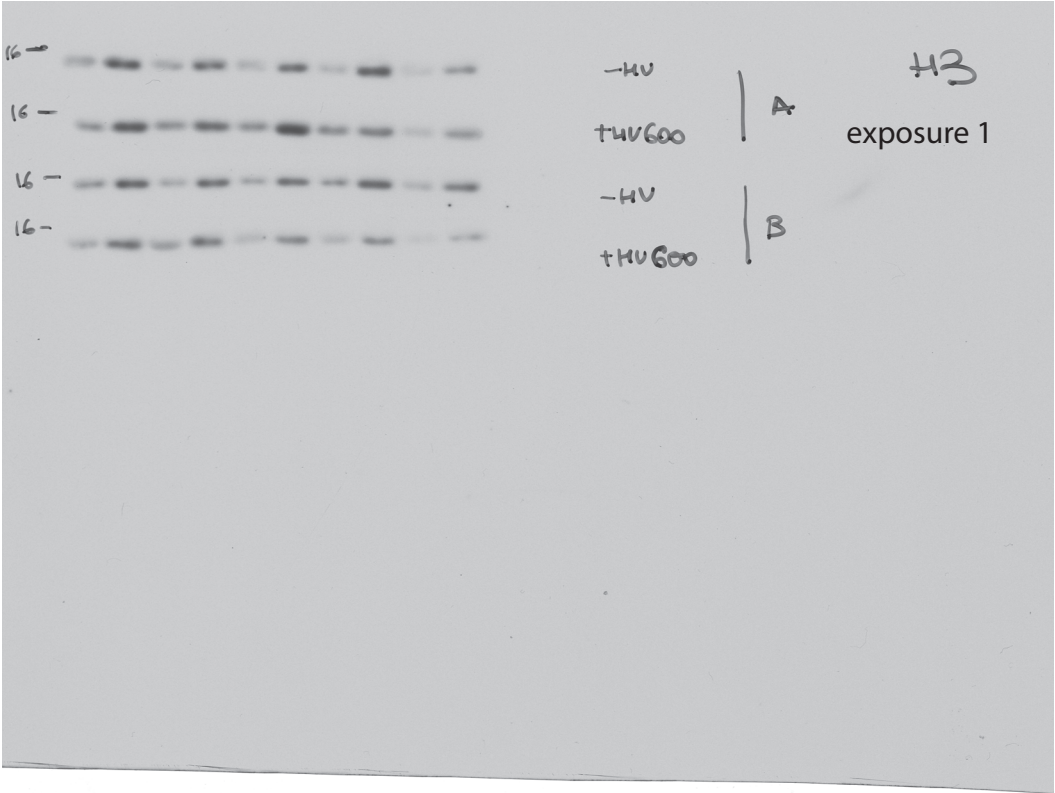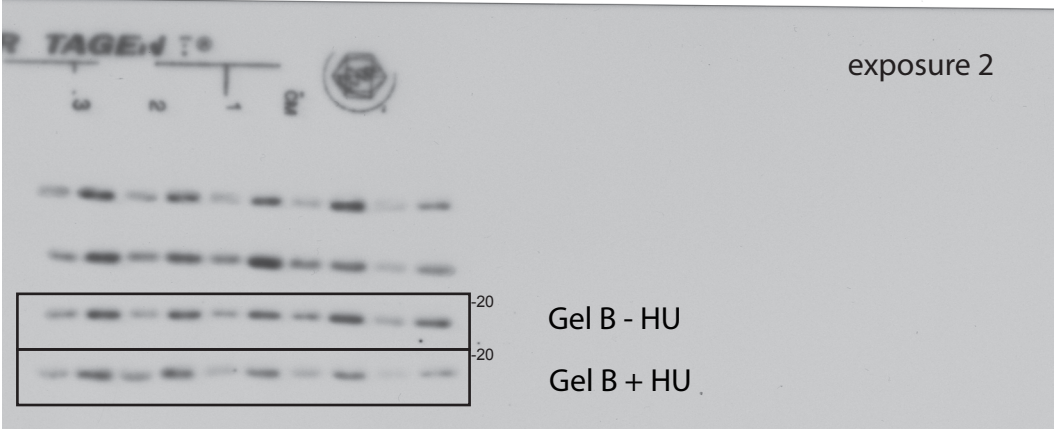

Supplement: Source Data Fig. 1 — Unprocessed western blots. [file 41556_2023_1167_MOESM7_ESM.pdf]

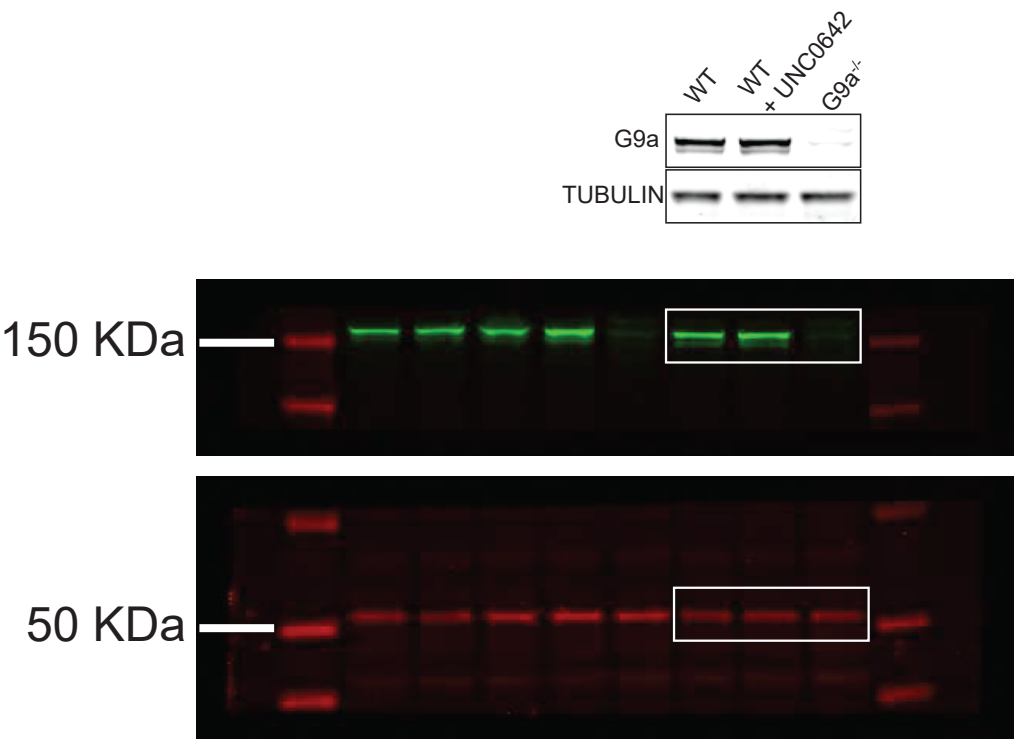

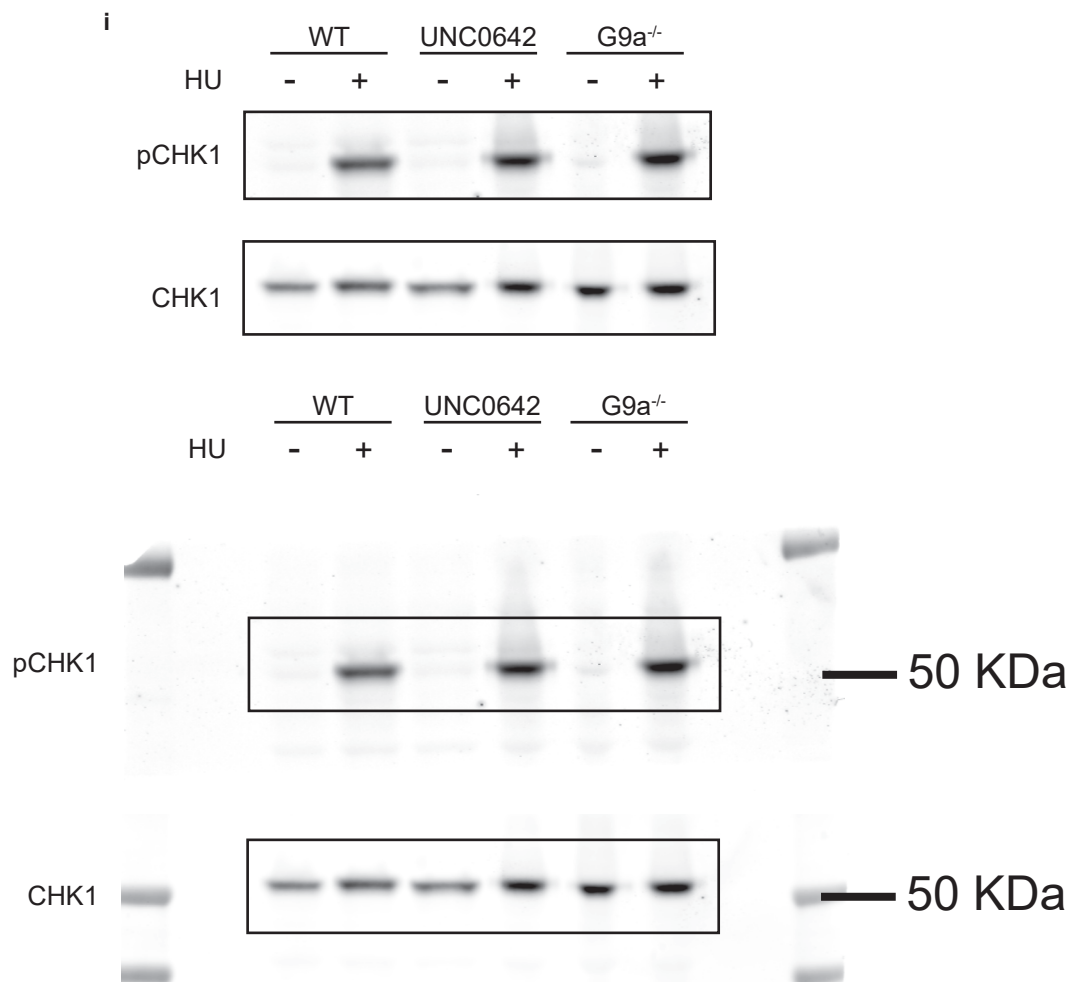

Supplement: Source Data Extended Data Fig./Table 4 — Unprocessed western blots. [file 41556_2023_1167_MOESM18_ESM.pdf]
