# Supplementary material for: Dynamic de novo heterochromatin assembly and disassembly at replication forks ensures fork stability
Source: Nat Cell Biol. 2023 Jul 6;25(7):1017–32. doi: 10.1038/s41556-023-01167-z (PMC10344782; doi:10.1038/s41556-023-01167-z)

Unprocessed western blots associated with **Extented data fig. 1c**

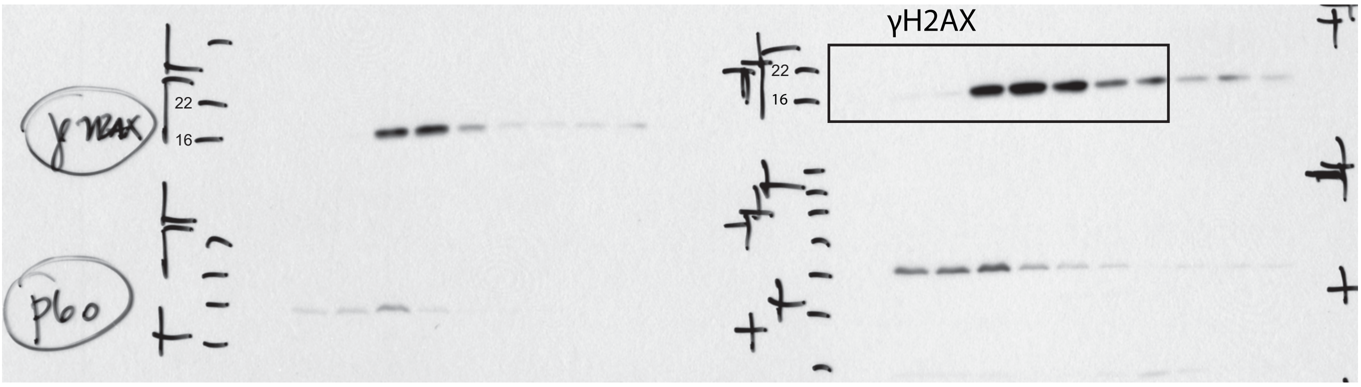

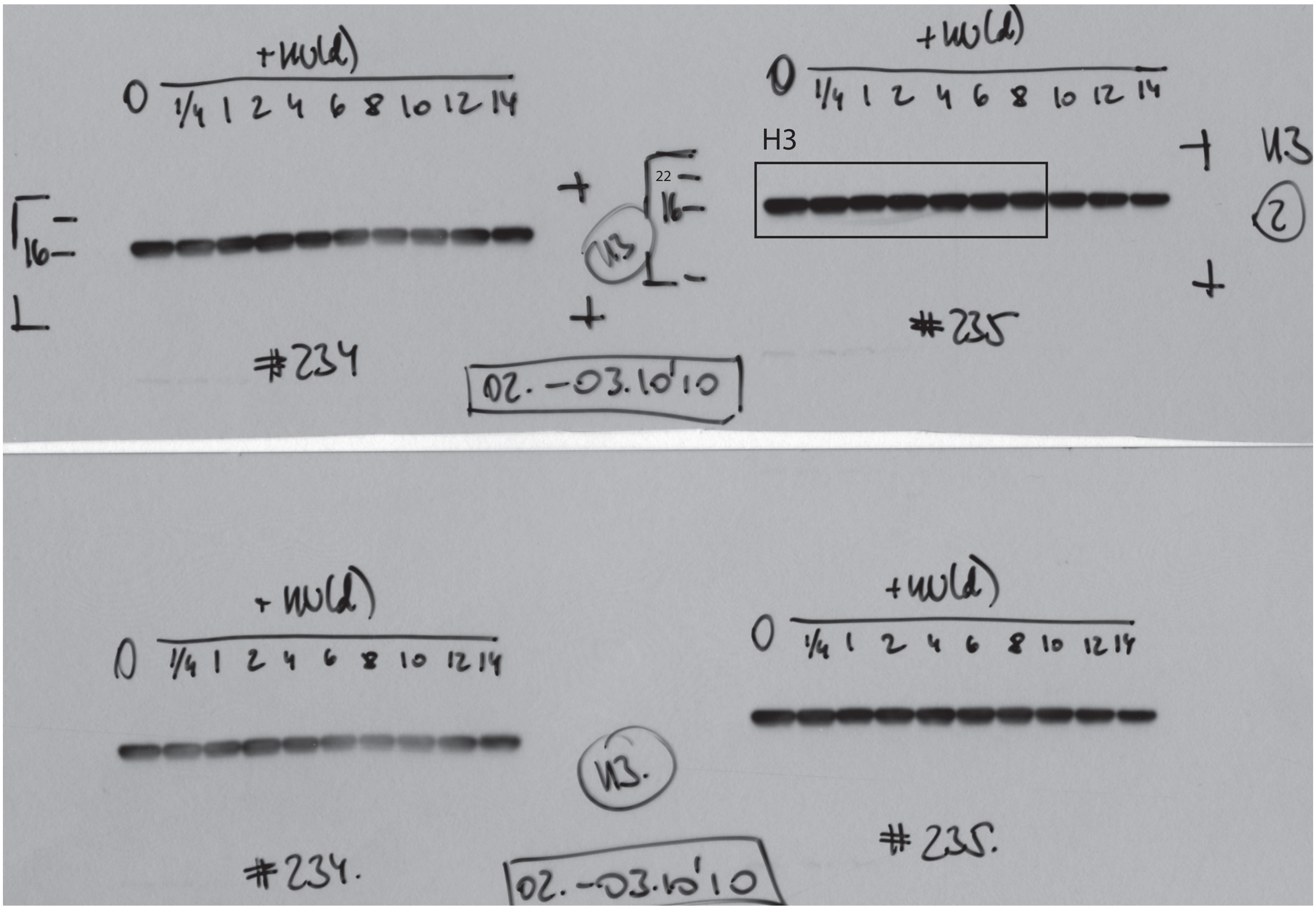

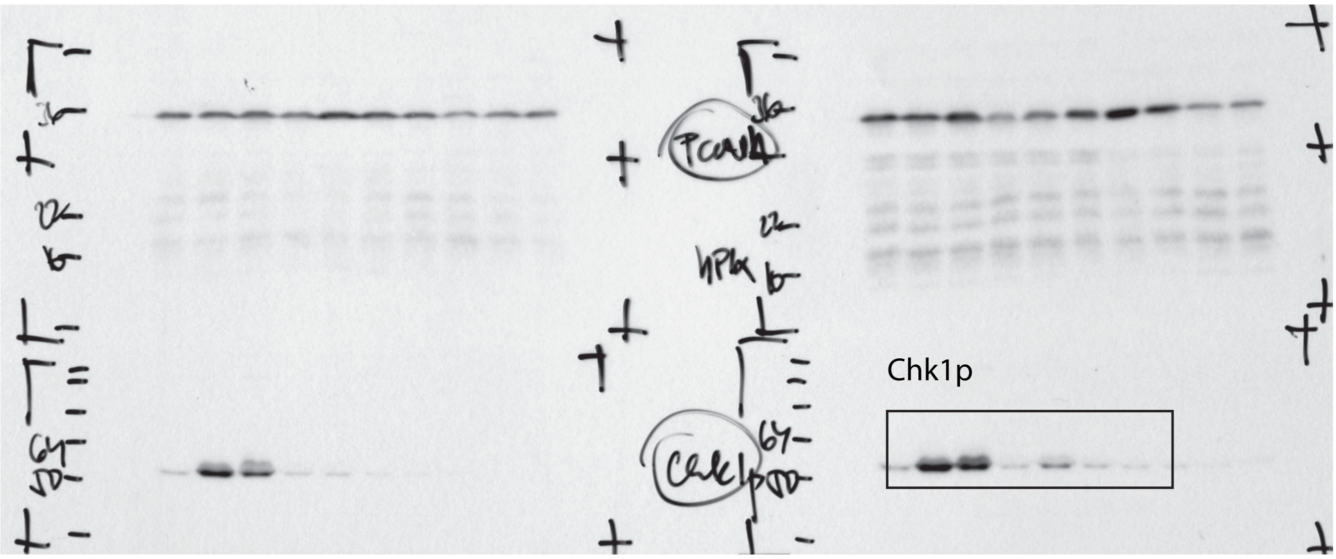

Unprocessed western blots associated with **Extented data fig. 1c**

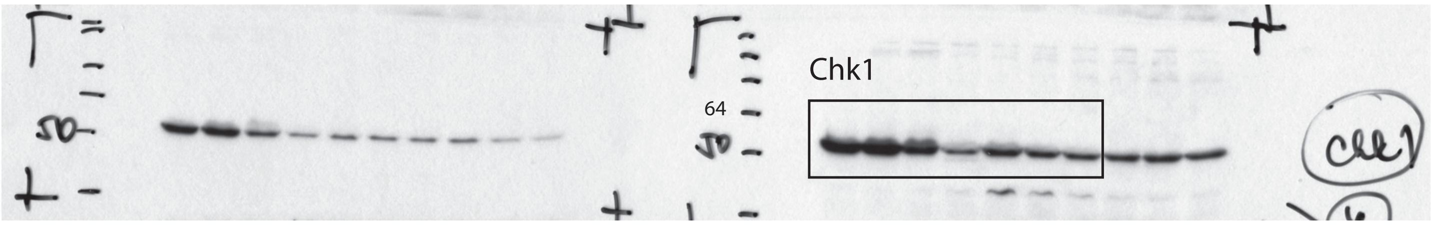

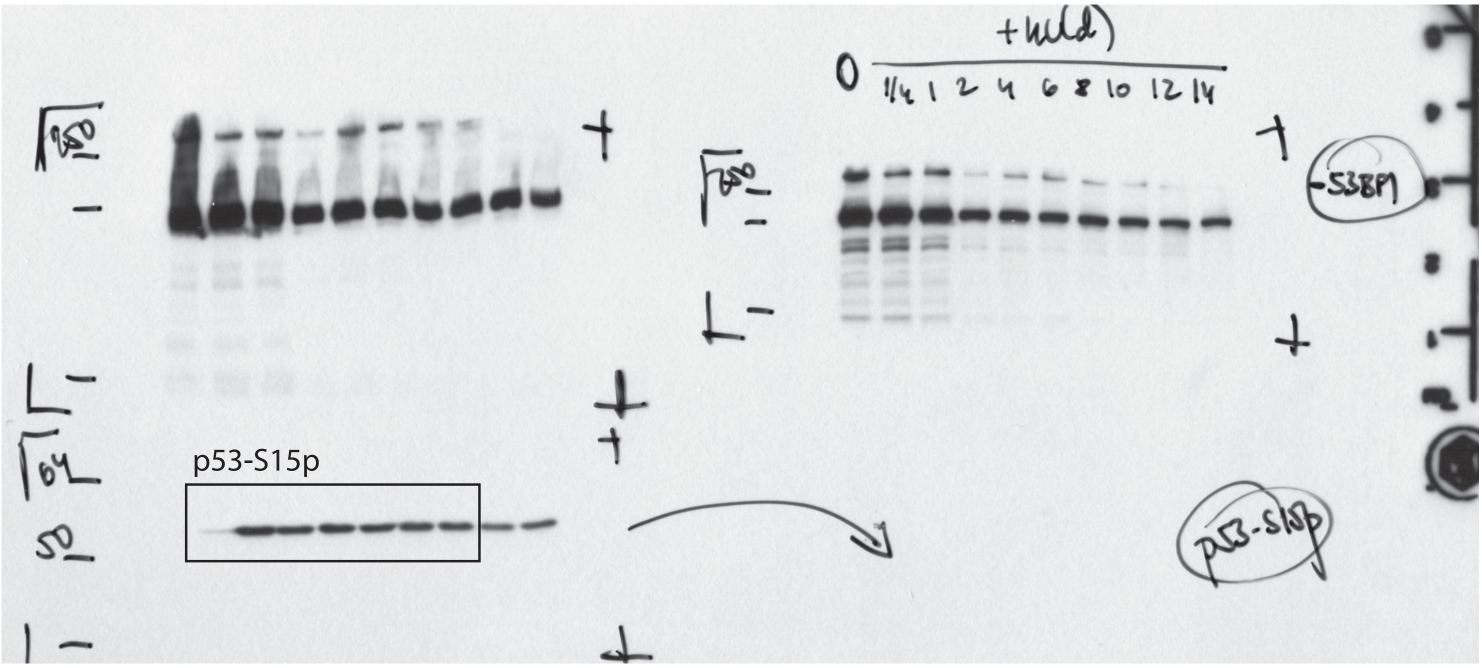

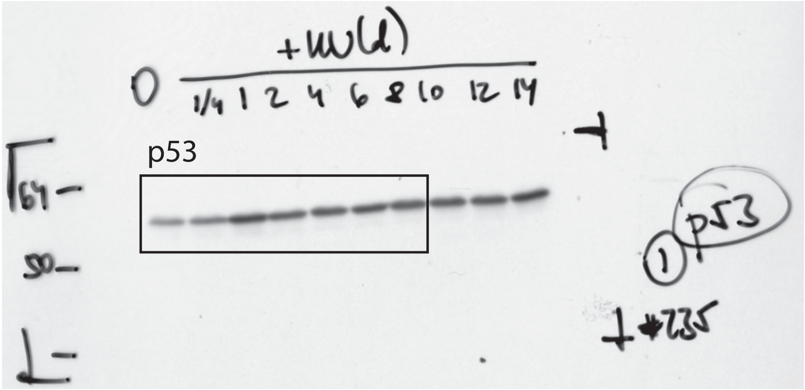

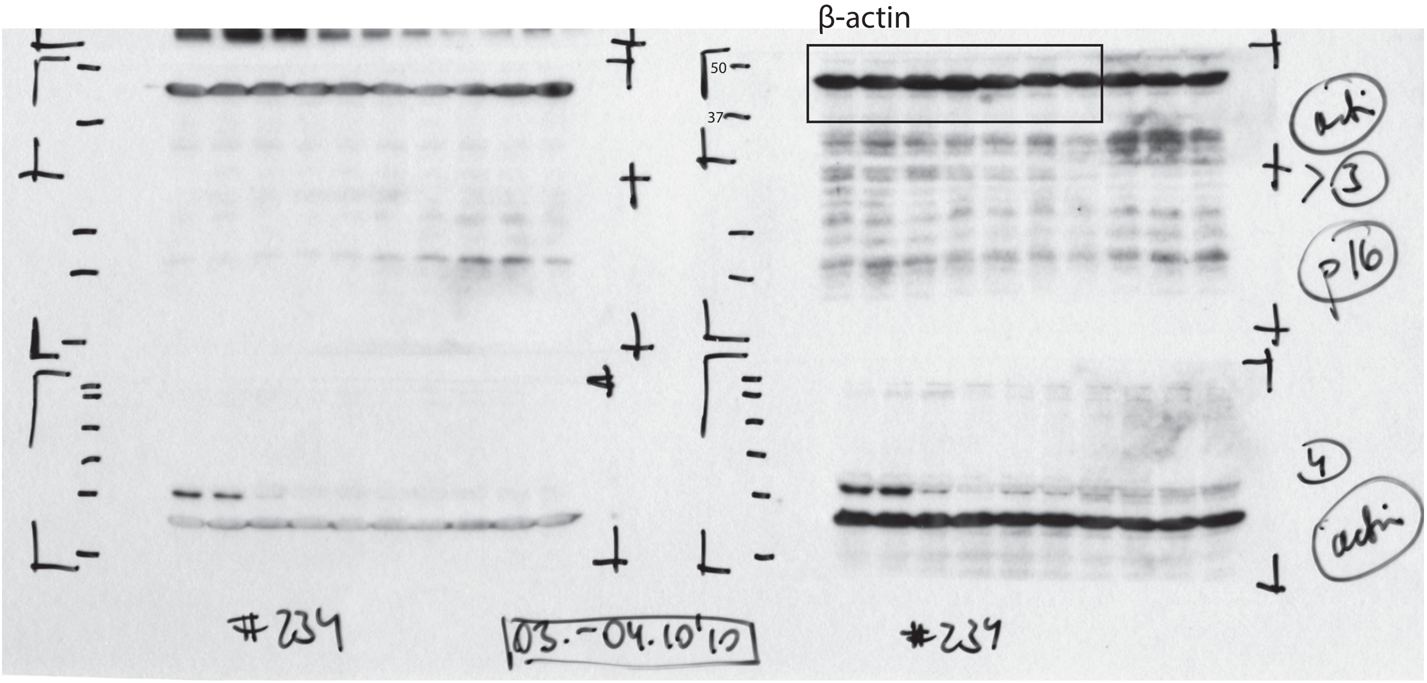

Supplement: Source Data Extended Data Fig./Table 1 — Unprocessed western blots. [file 41556_2023_1167_MOESM14_ESM.pdf]
